# Recurrent Malignant Pleural Effusion Management: Evaluating Quality Gaps, Outcomes, Costs, and Adherence to Guideline-Consistent Care

**DOI:** 10.1016/j.chpulm.2025.100227

**Published:** 2025-11-04

**Authors:** Michael J. Wright, Phuong H. Nguyen, Elliot D. Backer, Lara Goitein, David J. Feller-Kopman

**Affiliations:** a Department of Medicine, Dartmouth-Hitchcock Medical Center, Lebanon, NH; b Department of Medicine, Beth Israel Deaconess Medical Center, Boston, MA; c Section of Pulmonary and Critical Care Medicine, Dartmouth-Hitchcock Medical Center, Lebanon, NH

**Keywords:** definitive management, guideline recommendations, indwelling pleural catheter, malignant pleural effusion management, repeat thoracentesis, VATS decortication, VATS pleurodesis

## Abstract

**Background:**

Guidelines recommend early definitive management (indwelling pleural catheter, pleurodesis, decortication, or combination approach) for patients with symptomatic, rapidly recurrent malignant pleural effusion (MPE). This study evaluates health care utilization among patients who received early definitive management (EDM), defined as definitive management at first episode of rapidly recurrent MPE, compared with patients who do not (no early definitive management [NEDM]).

**Research Question:**

What percentage of patients with rapidly recurrent symptomatic MPE receive guideline-consistent EDM and are there differences in care outcomes?

**Study Design and Methods:**

This retrospective cohort study analyzed medical records from Dartmouth-Hitchcock Medical Center (a 500-bed primary teaching hospital) from 2018 to 2022. The cohort included patients with biopsy-proven MPE and rapid recurrence within 14 days of the initial thoracentesis, requiring a second pleural procedure. We compared the outcomes between patients who received EDM and those with NEDM.

**Results:**

A total of 133 patients met the inclusion criteria: confirmed malignancy on initial thoracentesis and rapid recurrence requiring repeat pleural procedure within 14 days. Of these, 52 patients (39%) received EDM, whereas 81 (61%) underwent repeat thoracentesis or chest tube drainage (NEDM). Compared with patients with NEDM, patients with EDM had fewer total pleural procedures (2.08 vs 3.51; *P* < .01), a lower rate of MPE-related emergency department visits (events per patient, 0.23 vs 1.31; *P* < .01), and a lower rate of MPE-related hospitalizations (events per patient, 0.23 vs 0.99; *P* < .01). A cost analysis of MPE-related hospitalizations revealed substantially lower MPE-related hospitalization costs for patients with EDM compared with patients with NEDM ($9,857 vs $39,497 per patient). No significant differences in survival were observed (*P* = .80).

**Interpretation:**

Less than one-half of eligible patients received guideline-consistent EDM for rapidly recurrent MPE. Those not receiving EDM required more health care resources, including pleural procedures, emergency department visits, and hospitalizations. These findings underscore the importance of EDM in reducing health care utilization and improving patient care outcomes.

## Graphical abstract

**Figure:**
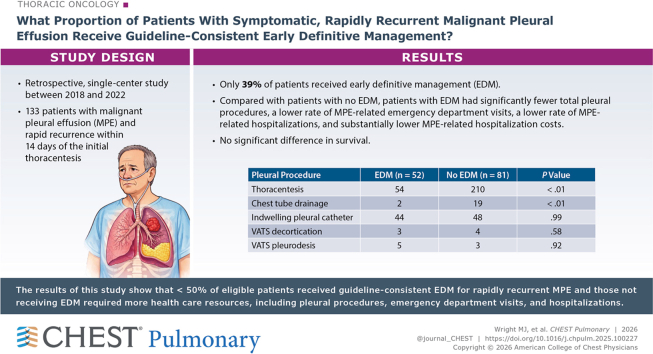

**Take-Home Points**

**Study Question:** What proportion of patients with symptomatic, rapidly recurrent malignant pleural effusion receive guideline-consistent early definitive management?

**Results:** Fewer than one-half of eligible patients received guideline-consistent early definitive management. Patients who did had fewer pleural procedures, emergency department visits, and hospitalizations.

**Interpretation:** These findings highlight substantial gaps in malignant pleural effusion management and emphasize the role of early definitive management in reducing health care utilization and improving patient outcomes.

Patients with malignant pleural effusion (MPE) have a poor prognosis with average life expectancy ranging from 3 to 12 months on diagnosis.^1^^,^^2^ It is estimated that > 150,000 cases of MPE are diagnosed yearly in the United States.^3^, ^4^, ^5^ MPE often leads to significant dyspnea and respiratory symptom burden at the end of life.^6^^,^^7^ Unfortunately, almost all MPEs that undergo pleural drainage will recur, with up to 58% of patients having a recurrence within 1 month of initial diagnostic thoracentesis.^8^^,^^9^ Managing MPE with serial thoracenteses is associated with an increased number of pleural procedures, pneumothoraxes, and emergency department (ED) procedures.^9^ The British Thoracic Society^2^ first recommended definitive management for symptomatic recurrent MPE in its 2010 guidelines. The American Thoracic Society^10^ subsequently endorsed a similar approach in its 2018 guidelines, recommending early consideration of indwelling pleural catheter (IPC), pleurodesis, surgical decortication, or combination therapy for patients with symptomatic, rapidly recurrent MPE. The 2023 British Thoracic Society^11^ pleural guidelines continue to endorse this approach.

Although guidelines exist for the management of patients with recurrent MPE, it remains unclear the extent to which clinical practices follow the outlined recommendations.^10^^,^^11^ Previous research based on a large Medicare database suggests that fewer than one-quarter of patients with MPE receive guideline-consistent care.^9^ This study seeks to add to that understanding by investigating adherence in a large academic medical center with a dedicated interventional pulmonology and interventional radiology services, which might be expected to have higher adherence than the national average. It also seeks to provide more detailed understanding of the health care resources required for guideline-consistent vs non-guideline-consistent care, including ED visits and hospital days specifically related to pleural procedures.

## Study Design and Methods

We performed a retrospective cohort analysis of patient charts queried from Epic Systems electronic medical records (EMR) at Dartmouth-Hitchcock Medical Center a 500-bed hospital which is the primary teaching hospital in the Dartmouth Health System. The EMR was queried for all patients who had a thoracentesis based on Current Procedural Terminology code and a documented cancer diagnosis based on International Classification of Diseases, 10th Edition, code. This project received institutional review board approval from the Dartmouth-Hitchcock institutional review board (approval No. STUDY02001898). The study was granted an exemption by the Dartmouth Health institutional review board.

The number of pleural procedures, hospitalizations, ED visits, and hospitalization costs were compared using Wilcoxon rank sum tests. ED visit hospitalization rates and hospitalization days per patient were compared using a 2-sample Poisson test. Survival was analyzed using Kaplan-Meier univariate analysis. Total cost per admission data was obtained from our finance department, which includes both direct costs (associated with direct patient care—medications, supplies, and staff), fixed costs (linen, dietary, and administration staff), and overhead (finance, human resources, and rent).

### Study Participants

The cohort included patients who had rapidly recurrent proven MPE between 2018 and 2022 on EMR review. Proven MPE was defined as a positive cytology from pleural fluid during thoracentesis. Rapidly recurrent is defined as the patient requiring a repeat pleural procedure (chest tube, thoracentesis, IPC, pleurodesis, or decortication) on the same side within 14 days of prior diagnostic thoracentesis.

### Definitions of Types of Care

We defined definitive management (DM) as the patient receiving IPC, pleurodesis, or surgical decortication. We defined early definitive management (EDM) as receiving DM as the first pleural procedure after diagnostic thoracentesis (consistent with guideline-directed care). We defined no early definitive management (NEDM) as any patient receiving repeat thoracentesis or chest tube drainage as the first repeat pleural procedure. NEDM was further subdivided into patients who eventually received DM (late definitive management [LDM]) and those who did not have any DM (never definitive management [NDM]). Biopsy-proven MPE is defined as a positive cytology in pleural fluid or pleural biopsy.

### Outcomes

The primary outcome was the proportion of patients who received EDM. Secondary outcomes included ordering location and proceduralist specialty for pleural procedures, total number of pleural procedures, ED visits for recurrent symptomatic MPE, hospitalizations and inpatient days for recurrent symptomatic MPE, total cost for hospital care, and survival. For ED visits and hospitalizations, patient charts were individually reviewed. ED and hospitalizations due to recurrent symptomatic MPE were included only if the chief complaint was shortness of breath or respiratory distress and the patient received an ipsilateral pleural procedure (thoracentesis, chest tube, or definitive pleural procedure) in the ED or during admission. We present costs of care as total direct and indirect costs, as calculated using a combination of standard and supply based costing for medications, supplies, ancillary services, and nursing activities in the EPSi system and activity-based costing using custom logic in our Health Catalyst data warehouse platform for operating room and other procedural activities.

## Results

### EDM vs NEDM

A total of 133 patients met the inclusion criteria: confirmed malignancy on initial thoracentesis with rapid recurrence requiring a repeat pleural procedure within 14 days. Patient characteristics are summarized in Table 1. There were no significant differences between groups; however, there was a trend toward more lymphoma as the cancer type in the NEDM group. There were 101 patients who underwent initial diagnostic thoracentesis in the ED or an inpatient service. Of the included patients, 81 (61%) underwent a second thoracentesis or chest tube drainage, whereas the remaining 52 (39%) of these early recurrers received DM as their second pleural procedure (EDM). Of the orders for second thoracenteses (representing the branchpoint for NEDM), most originated from the hospital medicine service (35%), followed by the hematology-oncology service (19%). Most of both initial diagnostic and second thoracenteses in our organization were performed by interventional radiology (73% and 82%, respectively). The pulmonary service performed a small portion of initial diagnostic and second thoracenteses (9% and 6%, respectively). Our interventional pulmonary service was established in late 2021 and performed a small portion of diagnostic and second thoracenteses (6% and 14%, respectively).

**Table 1:** Patient Cohort Characteristics

Variable	Total Patient Cohort (N = 133)	Patients With Early Definitive Management (n = 52)	Patients With No Early Definitive Management (n = 81)	*P* Value
Age of first thoracentesis, y				
Range	25-93	25-93	33-92	
Median	69	70	68.5	
Race				
White	132 (99.2)	51	81	
Unknown	1 (0.8)	1	0	
Sex^a^				.82
Male	74 (55.5)	32 (61.5)	42 (51.8)	
Female	59 (45.5)	20 (38.5)	39 (48.2)	
Cancer type^b^				
Non-small cell lung cancer	52 (39.1)	24 (46.2)	28 (34.7)	.69
Small-cell lung cancer	6 (4.5)	3 (50)	3 (3.7)	.24
Breast	17 (12.8)	7 (41.2)	10 (12.3)	.99
Ovarian	9 (6.8)	1 (1.9)	7 (8.6)	.11
Other solid	34 (25.6)	17 (32.7)	18 (22.2)	.18
Lymphoma	10 (9.8)	0 (0)	10 (12.3)	.054
Leukemia	5 (3.8)	0 (0)	5 (6.2)	.12

Data are presented as No. (%) or as otherwise indicated.

a Analyzed using χ^2^ test.

b Analyzed using Fisher exact test.

Among the 52 patients with EDM, 40 (77%) had their definitive pleural procedures completed during the same hospital admission as the initial diagnostic thoracentesis. The median time from the first thoracentesis to second pleural procedure in all patients was 7 days (interquartile range, 4-12 days). An additional 50 patients underwent LDM, meaning that 102 (76%) of all patients eventually underwent DM for recurrent effusion.

### Total Unilateral Pleural Procedures

**Figure 1:**
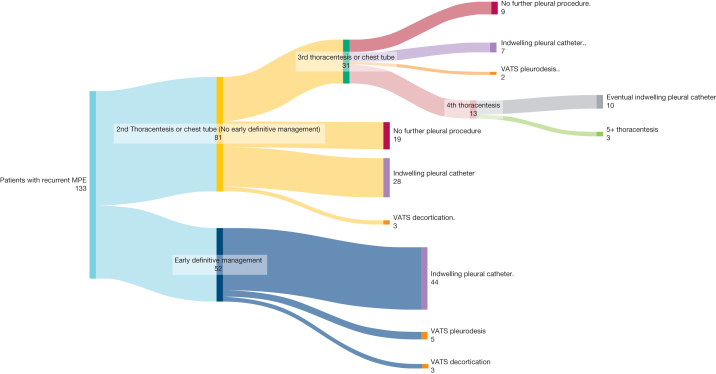
Sankey diagram showing the treatment pattern for patients in the cohort. No early definitive management is characterized as receiving repeat thoracentesis or chest tube as second pleural procedure. Late definitive management is any definitive pleural procedure after at least 3 thoracenteses. MPE = malignant pleural effusion; VATS = Video-Assisted Thoracoscopic Surgery.

Patients received 392 unilateral pleural procedures (thoracentesis, chest tube, and DM procedures), an average of 2.95 per patient (Table 2). There was a significant difference between the number of pleural procedures for those receiving EDM vs NEDM (2.08 vs 3.51; *P* < .001). Furthermore, there was also a significant difference in the mean number of pleural procedures between patients receiving EDM and patients receiving LDM (2.08 vs 3.80; *P* < .01). Patients who never received DM had a higher rate of thoracenteses than those with EDM (3.82 vs 1.04, respectively; *P* < .001). The pathway for patients receiving pleural procedures is shown in Figure 1.

**Table 2:** Total Number of Pleural Procedures

Pleural Procedure	EDM (n = 52)	NEDM (n = 81)	Total	*P* Value
Thoracentesis	54	210	273	< .01
Chest tube drainage	2	19	23	< .01
Indwelling pleural catheter	44	48	92	.99
VATS decortication	3	4	7	.58
VATS pleurodesis	5	3	8	.92
Total procedures	108	284	392	< .01

The number of procedures was compared using the Wilcoxon rank sum test. EDM = early definitive management; NEDM = no early definitive management; VATS = Video-Assisted Thoracoscopic Surgery.

Of the 102 patients receiving DM at any point, 8 (6%) subsequently required a total of 14 additional pleural procedures after DM (1.75 per patient). Four of these were in patients receiving EDM (0.08 per patient).

### ED Visits, Hospitalizations, and Costs

Table 3 shows health care resource utilization for patients with MPE. Of all 133 patients, there were a total of 242 ED visits within 6 months of initial diagnostic thoracentesis for any reason (1.82 visits per patient). Of these visits, 118 (49%) were due to MPE-related dyspnea, with patients receiving an ipsilateral pleural procedure (thoracentesis, tube thoracotomy, or DM) in either the ED or inpatient setting. There were 139 admissions to the hospital for any reason. Of these admissions, 92 (66%) were for MPE-related dyspnea requiring an ipsilateral pleural procedure. MPE-related admissions accounted for a total of 478 inpatient days.

**Table 3:** Health Care Utilization for Patients With Rapidly Recurrent MPE

Patient Population	Early Definitive Management (n = 52)	No Early Definitive Management (n = 81)	Total (N = 133)	*P* Value
Non-MPE ED visits (No., per patient rate)	43, 0.83	81, 1	124	.50
MPE-ED visits (No., per patient rate)	12, 0.23	106, 1.3	118	< .01
Non-MPE hospitalizations (No., per patient rate)	18, 0.35	29, 0.36	47	.97
MPE hospitalizations (No., per patient rate)	12, 0.23	80, 0.99	92	< .01
Average MPE hospitalization cost per admission	$42,713	$39,991	$40,346	.80
Average MPE hospitalization cost per patient	$9,857	$39,497	$27,908	< .01

Per patient rates of ED visits and hospitalizations were compared using a 2-sample Poisson test. Cost per admission and cost per patient were compared using the Wilcoxon rank sum test. ED = emergency department; MPE = malignant pleural effusion.

Patients who received EDM had a lower rate of MPE-related ED visits than patients with NEDM (events per patient, 0.23 vs 1.31; *P* < .01). This trend held true when comparing EDM with LDM (events per patient, 0.23 vs 1.64; *P* < .01) and EDM with NDM (events per patient, 0.23 vs 0.75; *P* < .01). Patients who received EDM also had a lower rate of MPE-related hospitalizations than patients with NEDM (events per patient, 0.23 vs 0.99; *P* < .01), with subgroups showing a similar trend: EDM vs LDM (events per patient, 0.23 vs 1.24; *P* < .01) and EDM vs NDM (0.23 vs 0.58; *P* < .01). There were also fewer total MPE-related inpatient days for EDM vs NEDM (days per patient, 1.90 vs 5.96; *P* < .01). Total direct cost of all MPE-related hospitalizations per patient was substantially less for EDM than for NEDM ($9,857 vs $39,497 per patient; *P* < .001), with subgroups showing a similar trend: LDM ($49,818 per patient) and NDM ($22,850 per patient). There was no statistical difference between individual hospitalization costs (*P* = .80).

### Survival

**Figure 2:**
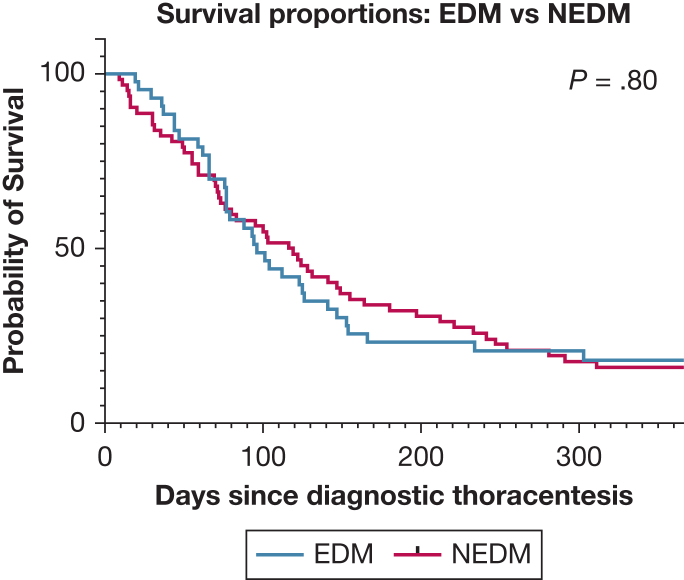
Kaplan-Meier survival curve comparing patients who received early definitive management vs no early definitive management. There was no significant difference in survival between groups; however, this study was not powered to evaluate survival. EDM = early definitive management; NEDM = no early definitive management.

Figure 2 shows the survival curve for patients who undergo EDM vs NEDM and LDM. There was no significant difference in survival between the groups (*P* = .80).

## Discussion

Our analysis highlights a significant gap in guideline-consistent care for the appropriate use of DM (IPC, pleurodesis, or surgical decortication) in patients with rapidly recurrent MPEs. According to guideline recommendations that have been in place since 2010, such patients should receive DM rather than repeat thoracentesis due to known improvement in patient focused outcomes such as dyspnea and interaction with the health care environment. We identified a high-risk patient group that had positive cytology on initial thoracentesis and rapid recurrence within 14 days, who should have been referred for early guideline-consistent DM. Within our hospital system, only 39% (n = 52) of these high-risk patients received guideline-consistent care with a definitive procedure as the second pleural procedure. A large proportion of the remaining patients ultimately received a definitive procedure (62%), but only after receiving what were possibly unnecessary procedures and hospital visits in the interim. Additionally, the pulmonary and interventional pulmonary service performed fewer repeat thoracenteses than interventional radiology. Since establishment in late 2021, the interventional pulmonology service performed most IPC placement (81%) during this time frame.

We found that among patients with MPE, MPE-related visits represented 49% of all ED visits and 66% of all inpatient admissions in the 6 months after the initial diagnostic procedure. This represents a large health care burden that could be reduced with EDM in this patient population. At our organization, patients not receiving guideline-consistent care required substantially more health care resources, requiring higher rates of pleural procedures, ED visits, and hospital visits. They had a higher number of inpatient days, and higher total costs from ED and hospital visits than their counterparts. In our organization, the average cost of MPE-related hospitalization per patient was significantly lower for patients receiving EDM than those with LDM or NDM. If all patients had received guideline-consistent care and had comparable MPE-related hospitalization costs with the EDM cohort, this could have saved approximately $29,640 per patient with NEDM. Survival curves did not differ substantially, but this study was not powered to determine survival differences.

Earlier research has similarly suggested a low rate of adherence to guideline-consistent care of patients with MPE. Ost et al^9^ used a large, national Medicare database to estimate a 24% rate of adherence. The slightly higher rate of adherence we found at our organization, 39%, may reflect the setting of a large academic medical center with a dedicated interventional radiology and interventional pulmonary service, and possibly a shift in practice patterns since 2007 to 2011. Ost et al^9^ also found higher use of health care resources among patients with MPE not receiving guideline-consistent care. Our study adds to this prior work by isolating ED and hospital admissions specifically related to MPE, for a more targeted estimate of health care resources specifically attributed to non-guideline-consistent care. We also report total direct cost per hospitalization. To our knowledge, this is the first study to estimate cost savings from guideline-consistent treatment of MPE and to use activity-based cost accounting rather than Medicare charges. Finally, we examined adherence in a large academic medical center setting.

The gap in guideline-consistent care for MPE revealed in this study is perhaps unsurprising, given the challenges inherent in implementation science, where integrating guidelines into clinical practice is difficult. For instance, the translation of ARDSnet data into clinical practice has taken more than a decade, despite robust mortality benefit with many large-scale studies continuing to highlight suboptimal adherence.^12^^,^^13^ The publication of guidelines alone is insufficient for ensuring implementation. Effective adherence requires a grassroots-level effort with introspective review of practice followed by implementation. The current study lays the groundwork for a quality improvement initiative to improve our organization’s treatment of patients with MPE and identifies key locations and specialties for targeted interventions. Moreover, it challenges the assumption that many implementation failures are substantially less prevalent in academic medical centers.

Our study has several limitations. One limitation is that our data set does not explore patient-oriented decision-making information. It is possible that some patients are being referred for DM in a timely manner but are opting for a conservative approach for any one of several reasons (eg, the desire to avoid more invasive procedures, care of indwelling catheters, insurance barriers). However, our data show that more than three-quarters of patients not receiving EDM will go on to eventually receive DM, suggesting that this population may be small. Our study is also a single-center study and contains a relatively small sample size, which limits its generalizability to other hospital systems and may introduce sampling error. To select patients with the strongest indication for definitive management, our study also only included patients with rapid recurrence within 14 days of initial diagnostic thoracentesis. It therefore does not provide insight into the clinical outcomes of patients who have recurrence beyond 14 days. The cohort also included 21 patients with potentially chemotherapy-sensitive malignancies (Non-small cell lung cancer [NSCLC], leukemia, and lymphoma). We individually reviewed each of these cases to assess candidacy for definitive management. All were found to have relapsed or advanced-stage disease, and in our judgment, would have been appropriate candidates for DM at first recurrence. Our cost analysis uses institutional total cost data, which includes both direct patient care and indirect costs, and although the exact costs may not be generalizable to other health care systems, it is likely other systems would see significant cost savings with EDM. Our analysis is also limited to ED and inpatient visits due to dyspnea requiring repeat pleural procedure. It is unclear whether patients with EDM have higher or lower rates of outpatient visits and corresponding costs. Additionally, our study does not include costs associated with DM (ie, infection of IPC, IPC bottles). Due to limitations in how our hospital system stores cost data, we were unable to calculate the cost associated with ED visits associated with an inpatient admission; thus, a cost analysis of ED visits was not included in this study and is not included in total cost analysis. It is likely that health care costs differ between hospitals and health systems, which may limit the generalizability of the findings. Finally, our study is not randomized, and the EDM and NEDM groups may differ in important ways that affect the outcomes we studied. It may be, for example, that patients with NEDM represent a sicker population, and that their higher resource use reflects this in addition to the direct effects of care pathways. However, the EDM and NEDM cohorts appeared similar in the variables measured, apart from more lymphoma as the primary cancer type in the NEDM group, which might have been expected to bias results in the opposite direction because lymphoma is a relatively treatable malignancy.

## Interpretation

We found a large gap in the use of guideline-consistent management of rapidly recurrent MPE in a large academic medical center, despite the presence of an interventional radiology and interventional pulmonology service committed to guideline-consistent care. Patients receiving multiple thoracenteses had a higher number of pleural procedures, MPE-related ED visits and hospitalizations, and inpatient days, and total higher admission costs. The findings of this study provide the foundation for quality improvement in our own organization and perhaps grounds for similar investigations in others.

## Funding/Support

The authors have reported to *CHEST Pulmonary* that no funding was received for this study.

## Financial/Nonfinancial Disclosures

None declared.
